# Relationships of drinking and smoking with peripheral arterial stiffness in Chinese community-dwelling population without symptomatic peripheral arterial disease

**DOI:** 10.1186/s12971-017-0144-9

**Published:** 2017-10-25

**Authors:** Shihui Fu, Qixian Wu, Leiming Luo, Ping Ye

**Affiliations:** 1 Department of Geriatric Cardiology, Chinese People’s Liberation Army General Hospital, Beijing, 100853 China; 2 Department of Cardiology and Hainan Branch, Chinese People’s Liberation Army General Hospital, Beijing, China; 3 Department of Nephrology and Hainan Branch, Chinese People’s Liberation Army General Hospital, Beijing, China

**Keywords:** Alcohol drinking, Chinese community-dwelling population, Cigarette smoking, Peripheral arterial disease, Peripheral arterial stiffness

## Abstract

**Background:**

Peripheral arterial stiffness gives rise to the high prevalence of peripheral arterial disease (PAD). It is necessary to conduct a large-scale study in Chinese community-dwelling population to clarify the relationships of alcohol and tobacco consumption with peripheral arterial stiffness. Most studies had a small sample size, and were not performed in Chinese community-dwelling population without symptomatic PAD. This analysis was designed to examine the relationships of alcohol and tobacco consumption with peripheral arterial stiffness in Chinese community-dwelling population without symptomatic PAD.

**Methods:**

In a large health check-up program in Beijing (2007–2009), 2624 participants were involved in this analysis, and carotid-radial pulse wave velocity (crPWV) was measured following standard procedure. Physical examinations were performed by well-trained physicians. Blood samples were analyzed by qualified technicians in central laboratory. Initially, either alcohol drinking or cigarette smoking, and then both alcohol drinking and cigarette smoking, were put in one model of multivariate Logistic regression analyses.

**Results:**

Median age was 54 years, and median value of crPWV was 9.4 m/s; 51.8% were males, 27.6% were smokers and 30.6% were drinkers. In Logistic regression analyses with either alcohol drinking or cigarette smoking, and both alcohol drinking and cigarette smoking, in one model, cigarette smoking was independently associated with crPWV (*P* < 0.05 for all), and alcohol drinking was not independently associated with crPWV (*P* > 0.05 for all).

**Conclusions:**

Cigarette smoking had an independent relationship with peripheral artery stiffness, and there was no independent relationship between alcohol drinking and peripheral arterial stiffness in Chinese community-dwelling population without symptomatic PAD.

## Background

Peripheral arterial stiffness gives rise to the high prevalence of peripheral arterial disease (PAD) [1, 2]. Carotid-radial pulse wave velocity (crPWV) is generally applied as a parameter of peripheral arterial stiffness [3]. Limited studies have discussed the relationship between alcohol consumption and peripheral arterial stiffness [4–6]. Although some studies have found that alcohol consumption decreases the peripheral arterial stiffness, other studies have suggested that alcohol consumption is closely associated with increased peripheral arterial stiffness [5, 6]. Meanwhile, another study has reported that alcohol consumption is not associated with peripheral arterial stiffness [7]. Moreover, most studies had a small sample size, and were not performed in Chinese community-dwelling population without symptomatic PAD. Therefore, it is necessary to conduct a large-scale study in Chinese community-dwelling population to clarify the relationship between alcohol consumption and peripheral arterial stiffness.

In addition, although previous studies have shown that tobacco use is prone to increase peripheral arterial stiffness, other studies have found no significant difference in peripheral arterial stiffness between nonsmokers and smokers [8–10]. To evaluate the relationship between smoking and peripheral arterial stiffness through large-scale studies in Chinese community-dwelling population without symptomatic PAD may provide more important information. To sum up, the current analysis was designed to examine the relationships of alcohol and tobacco consumption with peripheral arterial stiffness in Chinese community-dwelling population without symptomatic PAD.

## Methods

### Study population

This large health check-up program was performed in Beijing, China, from May 2007 to July 2009. A stratified cluster sampling design was applied in this survey. In the first stage of sampling, three districts (Fengtai, Shijingshan and Daxing) were selected from 18 districts in Beijing. In the second stage of sampling, four communities were selected from these districts. In the third stage of sampling, community-dwelling residents were selected from these communities. Each one was Han Chinese and older than 18 years. Symptomatic PAD was diagnosed by chief physicians based on the existing symptoms (intermittent claudication and rest pain) and positive examinations (color doppler ultrasound, computed tomography angiography, magnetic resonance angiography and digital subtraction angiography). After excluding 165 participants with symptomatic PAD, 2624 participants were involved in the final analysis. The study protocol was approved by Ethics Committee of Chinese People’s Liberation Army General Hospital, China. Each participant provided written informed consent to be included in the study.

### General survey and physical examination

All anthropometries were measured by well-trained physicians. Weight was measured on a digital scale while wearing light clothing and no shoes. Height was measured on standing participants without shoes using a wall-mounted measuring tape. Body mass index (BMI) was calculated as weight (kg) divided by height squared (m^2^). Waist circumference (WC) was measured on standing participants with a soft tape midway between the lowest rib and iliac crest. Blood pressure was measured with a standard mercury sphygmomanometer using the first and fifth Korotkoff sounds as systolic and diastolic blood pressure in the sedentary position while participants had rested for at least 5 min. Appropriate size of cuff was based on arm circumference. Pulse pressure (PP) is the difference between systolic and diastolic blood pressure. Cigarette smoking was defined as the consumption of one or more cigarettes per day for at least 1 year [11]. Alcohol drinking was defined as the consumption of 30 g or more alcohols per week for at least 1 year [12].

### Peripheral arterial stiffness

Peripheral arterial stiffness was assessed by automatic crPWV measurement using the Complior Colson device (Createch Industrie, Garges les Gonesse, France) in the morning, in a quiet environment, at stable temperature; the technical characteristics of this device have been described previously [13]. Participants were studied in the supine position after resting for at least 5 min. crPWV along the artery was measured by using two strain-gauge transducers (TY-306 Fukuda pressure-sensitive transducer [Fukuda Denshi Co., Tokyo, Japan]) fixed transcutaneously over the course of arteries separated by a known distance; the carotid and radial arteries (all on the right side) were used. After pulse waveform was recorded, digitization process was initiated by the operator, and automatic calculation of time delay between two upstrokes was started. Measurements were repeated over 10 different cardiac cycles, and mean value was used for the final analysis. crPWV was calculated from the measurement of pulse transit time and the distance traveled by the pulse between two recording sites (measured on the surface of body in meters), according to the following formula: crPWV (m/s) = distance (m)/transit time (s).

### Laboratory test

Blood samples were collected between 8 am and 10 am after participants fasting at least 12 h, and delivered to the central laboratory in Department of Biochemistry on the same day. Fasting blood glucose (FBG), high density lipoprotein cholesterol (HDL-c) and low density lipoprotein cholesterol (LDL-c) were determined using the enzymatic assays (Roche Products Ltd., Basel, Switzerland) on a full automatic biochemical autoanalyzer (COBAS 6000; Roche Products Ltd.). All qualified technicians were blinded to the clinical data of participants.

### Statistical analysis

Categorical variables were expressed as number (percentage). Continuous variables were expressed as mean (standard deviation) for data with a normal distribution and median (interquartile range) for non-normally distributed data. Participants were stratified by the status of smoking and drinking. Statistical comparison was made between groups using Student’s t-test for continuous variables (normal distribution), Mann–Whitney U test for continuous variables (skewed distribution) and chi-square analysis for categorical variables. Logistic regression analysis was performed following three models: unjusted model, in the first model with adjustment of age and gender, and in the second model with adjustment of age, gender, BMI, WC, PP, FBG, HDL-c and LDL-c. Initially, either alcohol drinking or cigarette smoking was put in one model, and then both alcohol drinking and cigarette smoking were put in one model. All analyses were conducted using Statistical Package for Social Science version 17 (SPSS Inc., Chicago, IL, USA). *P* < 0.05 was considered statistically significant.

## Results

Median age of all participants was 54 years with an age range of 18 to 96 years; 51.8% were males, 27.6% were smokers and 30.6% were drinkers. Median value of crPWV was 9.4 m/s. Table 1 presented the demographic and clinical characteristics of all participants stratified by the status of smoking and drinking. Compared with nonsmokers, smokers had significantly higher crPWV, BMI and WC levels, and lower HDL-c level (*P* < 0.05 for all). Percentages of males and drinkers were significantly higher in smokers than nonsmokers (*P* < 0.001 for all). Compared with nondrinkers, drinkers were younger, with significantly higher crPWV, BMI and WC levels, and lower PP, FBG, LDL-c and HDL-c levels (*P* < 0.05 for all). Percentages of males and smokers were significantly higher in drinkers than nondrinkers (P < 0.001 for all). In Logistic regression analyses with either alcohol drinking or cigarette smoking in one model (Table 2), cigarette smoking was independently associated with crPWV in all three models (*P* < 0.05 for all), and alcohol drinking was associated with cfPWV in the unjusted model (*P* < 0.05), but not independently associated with them in the first and second models (*P* > 0.05 for all). In Logistic regression analyses with both alcohol drinking and cigarette smoking in one model, cigarette smoking was independently associated with crPWV in all three models (*P* < 0.05), and alcohol drinking was associated with cfPWV in the unjusted model (P < 0.05), but not independently associated with them in the first and second models (P > 0.05 for all).

**Table 1:** Demographic and clinical characteristics of participants stratified by the status of smoking and drinking in a Beijing community-dwelling population (2007–2009)

Characteristics	Nonsmokers (*n* = 1901)	Smokers (*n* = 723)	*P* value^a^	Nondrinkers (*n* = 1821)	Drinkers (*n* = 803)	*P* value^b^
Age (year)	54(40–66)	54(39–67)	0.955	58(47–68)	42(32–57)	<0.001
Males (%)	729(38.3)	629(87.0)	<0.001	607(33.3)	751(93.5)	<0.001
BMI (kg/m^2^)	24.98(22.72–27.25)	25.39(23.32–27.52)	0.002	24.98(22.67–27.39)	25.39(23.46–27.18)	0.015
WC (cm)	84(77–91)	89(83–95)	<0.001	85(77–92)	87(81–93)	<0.001
Alcohol drinking (%)	391(20.6)	412(57.0)	<0.001			
Cigarette smoking (%)				311(17.1)	412(51.3)	<0.001
PP (mmHg)	48(40–60)	48(40–58)	0.427	50(40–60)	46(40–54)	<0.001
FBG (mmol/L)	4.92(4.57–5.39)	4.95(4.55–5.47)	0.513	4.95(4.56–5.49)	4.90(4.59–5.25)	0.014
LDL-c (mmol/L)	2.75(2.25–3.25)	2.76(2.29–3.22)	0.962	2.85(2.34–3.35)	2.54(2.11–3.01)	<0.001
HDL-c (mmol/L)	1.38(1.18–1.63)	1.26(1.06–1.48)	<0.001	1.36(1.16–1.62)	1.31(1.10–1.53)	<0.001
crPWV (m/s)	9.2(8.4–10.0)	9.8(9.1–10.9)	0.001	9.2(8.3–10.0)	9.8(9.0–10.9)	0.008

Notes: ^a^ *P* value for difference between nonsmokers and smokers; ^b^ *P* value for difference between nondrinkers and drinkers

*Abbreviations*: *BMI* Body mass index, *WC* Waist circumference, *PP* Pulse pressure, *FBG* Fasting blood glucose, *LDL-c* Low density lipoprotein cholesterol, *HDL-c* High density lipoprotein cholesterol, *crPWV* carotid-radial pulse wave velocity

**Table 2:** Multivariate relationships of carotid-radial pulse wave velocity with cigarette smoking and alcohol drinking in a Beijing community-dwelling population (2007–2009)

Characteristics		crPWV (m/s)	
		OR value (95% CI)	*P* value
Only one in models^a^			
Alcohol drinking	Unjusted model	2.299(1.936–2.729)	<0.001
	1st model^c^	1.227(0.989–1.521)	0.063
	2nd model^d^	1.209(0.973–1.502)	0.087
Cigarette smoking	Unjusted model	2.291(1.918–2.737)	<0.001
	1st model^c^	1.343(1.099–1.641)	0.004
	2nd model^d^	1.293(1.056–1.584)	0.013
Both two in models^b^			
Alcohol drinking	Unjusted model	1.892(1.577–2.270)	<0.001
Cigarette smoking	Unjusted model	1.845(1.528–2.229)	<0.001
Alcohol drinking	1st model^c^	1.163(0.934–1.449)	0.177
Cigarette smoking	1st model^c^	1.308(1.067–1.605)	0.010
Alcohol drinking	2nd model^d^	1.154(0.925–1.440)	0.204
Cigarette smoking	2nd model^d^	1.262(1.027–1.551)	0.027

Notes: ^a^Either alcohol drinking or cigarette smoking in one model; ^b^Both alcohol drinking and cigarette smoking in one model; ^c^First model: adjusted by age and gender; ^d^Second model: adjusted by age, gender, body mass index, waist circumference, pulse pressure, fasting blood glucose, high-density lipoprotein-cholesterol and low-density lipoprotein-cholesterol

*Abbreviations*: *crPWV* carotid-radial pulse wave velocity, *OR* Odds ratio, *CI* Confidence interval

## Discussion

The current analysis had the following key findings in Chinese community-dwelling population without symptomatic PAD: 1) there was an independent relationship between cigarette smoking and peripheral artery stiffness; 2) alcohol drinking had no independent relationship with peripheral arterial stiffness. In order to avoid the interaction between cigarette smoking and alcohol drinking, either alcohol drinking or cigarette smoking, and then both alcohol drinking and cigarette smoking, were put in one model of multivariate Logistic regression analyses. As the most important social and clinical implication of this analysis, to reduce tobacco use by taking every feasible measures could have potential to effectively delay the progression of peripheral arterial stiffness and avoid the development of PAD.

Peripheral arterial stiffness is closely related to the development of PAD.^1–3^ Although some studies have considered that alcohol use is an important risk factor associated with the development of peripheral arterial stiffness, other studies have revealed that alcohol use plays a significant role in the alleviation of peripheral arterial stiffness [5, 6]. In addition, alcohol use has been shown to have no influence on peripheral arterial stiffness [7]. The beneficial effect of alcohol use has been believed to be mainly due to its relationships with glucose-lipid metabolism and coagulation-fibrinolysis system, whereas the harmful effect of alcohol use is mainly due to its relationship with pulse pressure [14]. The current analysis proposed that alcohol intake had no independent relationship with peripheral arterial stiffness in Chinese community-dwelling population without symptomatic PAD.

Some studies have manifested that smoking leads to a significant increase in peripheral arterial stiffness [8, 9]. Smoking alters arterial structure and function through several mechanisms, such as endothelial dysfunction and lipid abnormalities [15]. However, there has been controversial results about effect of smoking on peripheral arterial stiffness in previous studies [8–10]. Moreover, little is known in Chinese community-dwelling population without symptomatic PAD about the relationship between tobacco use and peripheral arterial stiffness, and it is of great significance to know whether smoking affects the progression of peripheral arterial stiffness in this population [8, 9]. The current analysis provided evidence that there was an independent relationship between smoking and peripheral artery stiffness in Chinese community-dwelling population without symptomatic PAD.

The current analysis had some limitations. Firstly, the current analysis was cross-sectional, which limited inference. Secondly, floating population did not participate in the current analysis, and it was difficult to state the number of floating population. Thirdly, ‘healthy smoker’ and ‘healthy drinker’ effects could not be completely eliminated in the current analysis. Meanwhile, its strengths included a relatively large sample size and a range of biological measures.

## Conclusions

The current analysis demonstrated that cigarette smoking had an independent relationship with peripheral artery stiffness, and there was no independent relationship between alcohol drinking and peripheral arterial stiffness, in Chinese community-dwelling population without symptomatic PAD.

## Abbreviations

BMI: Body mass index
crPWV: Carotid-radial pulse wave velocity
FBG: Fasting blood glucose
HDL-c: High density lipoprotein cholesterol
LDL-c: Low density lipoprotein cholesterol
PAD: Peripheral arterial disease
WC: Waist circumference

## References

[1] Laurent S, Boutouyrie P, Asmar R (2001). Aortic stiffness is an independent predictor of all-cause and cardiovascular mortality in hypertensive patients. Hypertension.

[2] O’Rourke MF, Franklin SS (2006). Arterial stiffness: reflections on the arterial pulse. Eur Heart J.

[3] Lehmann ED (1999). Clinical value of aortic pulse-wave velocity measurement. Lancet.

[4] Nakanishi N, Kawashimo H, Nakamura K (2001). Association of alcohol consumption with increase in aortic stiffness: a 9-year longitudinal study in middle-aged Japanese men. Ind Health.

[5] Sierksma A, Muller M, van der Schouw YT (2004). Alcohol consumption and arterial stiffness in men. J Hyperten.

[6] Nakanishi N, Yoshida H, Kawashimo H (2001). Alcohol consumption and risk for increased aortic pulse wave velocity in middle-aged Japanese men. Angiology.

[7] Nakanishi N, Tatara K, Suzuki K (1998). Risk factors for the incidence of aortic stiffness by serial aortic pulse wave velocity measurement in middle-aged Japanese men. Environ Health Prev Med.

[8] Rhee MY, Na SH, Kim YK (2007). Acute effects of cigarette smoking on arterial stiffness and blood pressure in male smokers with hypertension. Am J Hypertens.

[9] Kubozono T, Miyata M, Ueyama K (2011). Acute and chronic effects of smoking on arterial stiffness. Circ J.

[10] Doonan RJ, Hausvater A, Scallan C (2010). The effect of smoking on arterial stiffness. Hypertens Res.

[11] Zhu Q, Xiao W, Bai Y (2016). The prognostic value of the plasma N-terminal pro-brain natriuretic peptide level on all-cause death and major cardiovascular events in a community-based population. Clin Interv Aging.

[12] Yang SH, Dou KF, Song WJ (2010). Prevalence of diabetes among men and women in China. N Engl J Med.

[13] Asmar R, Benetos A, Topouchian J (1995). Assessment of arterial distensibility by automatic pulse wave velocity measurement.Validation and clinical application studies. Hypertension.

[14] Wakabayashi I, Kobaba-Wakabayashi R, Masuda H (2002). Relation of drinking alcohol to atherosclerotic risk in type 2 diabetes. Diabetes Care.

[15] Ambrose JA, Barua RS (2004). The pathophysiology of cigarette smoking and cardiovascular disease: an update. J Am Coll Cardiol.

